# Expanding the genetic landscape of oral‐facial‐digital syndrome with two novel genes

**DOI:** 10.1002/ajmg.a.62337

**Published:** 2021-06-15

**Authors:** Alanna Strong, Laurie Simone, Anthony Krentz, Courtney Vaccaro, Deborah Watson, Hayley Ron, Jennifer M. Kalish, Helio F. Pedro, Elaine H. Zackai, Hakon Hakonarson

**Affiliations:** ^1^ Division of Human Genetics Children's Hospital of Philadelphia Philadelphia Pennsylvania USA; ^2^ The Center for Applied Genomics Children's Hospital of Philadelphia Philadelphia Pennsylvania USA; ^3^ Center for Genetic and Genomic Medicine Hackensack University Medical Center Hackensack New Jersey USA; ^4^ PreventionGenetics Marshfield Wisconsin USA; ^5^ Department of Pediatrics, Perelman School of Medicine University of Pennsylvania Philadelphia Pennsylvania USA; ^6^ Division of Pulmonary Medicine Children's Hospital of Philadelphia Philadelphia Pennsylvania USA

**Keywords:** *CEP164*, ciliopathy, oral‐facial‐digital syndrome, *TOPORS*

## Abstract

Oral‐facial‐digital syndromes (OFDS) are a heterogeneous and rare group of Mendelian disorders characterized by developmental abnormalities of the oral cavity, face, and digits caused by dysfunction of the primary cilium, a mechanosensory organelle that exists atop most cell types that facilitates organ patterning and growth. OFDS is inherited both in an X‐linked dominant, X‐linked recessive, and autosomal recessive manner. Importantly, though many of the causal genes for OFDS have been identified, up to 40% of OFD syndromes are of unknown genetic basis. Here we describe three children with classical presentations of OFDS including lingual hamartomas, polydactyly, and characteristic facial features found by exome sequencing to harbor variants in causal genes not previously associated with OFDS. We describe a female with hypothalamic hamartoma, urogenital sinus, polysyndactyly, and multiple lingual hamartomas consistent with OFDVI with biallelic pathogenic variants in *CEP164*, a gene associated with ciliopathy‐spectrum disease, but never before with OFDS. We additionally describe two unrelated probands with postaxial polydactyly, multiple lingual hamartomas, and dysmorphic features both found to be homozygous for an identical *TOPORS* missense variant, c.29 C>A; (p.Pro10Gln). Heterozygous *TOPORS* pathogenic gene variants are associated with autosomal dominant retinitis pigmentosa, but never before with syndromic ciliopathy. Of note, both probands are of Dominican ancestry, suggesting a possible founder allele.

## 1 INTRODUCTION

Ciliopathy syndromes are rare Mendelian disorders caused by dysfunction of the primary cilium, a mechanosensory organelle that exists atop most cell types that facilitates proper organ patterning and growth (Berbari et al., 2009; Fry et al., 2014). Oral‐facial‐digital syndromes (OFDS) represent a heterogenous group of ciliopathies, characterized by the core features of oral cavity malformations, such as tongue hamartomas, cleft palate, lobulated tongue, and hyperplastic frenula, craniofacial dysmorphisms such as down‐slanted palpebral fissures, hypertelorism, and broad and flat nasal bridge, and digit malformations such as brachydactyly, polydactyly, and syndactyly (Gurrieri et al., 2007). Affected individuals can also have mild to severe intellectual impairment, structural brain differences, hypothalamic hamartoma, facial milia, coloboma, retinopathy, missing, extra or defective teeth, clinodactyly, structural heart differences, and polycystic kidneys (Bruel et al., 2017; Franco & Thauvin‐Robinet, 2016). Importantly, features of OFDS can be seen in other ciliopathy syndromes, most commonly Joubert syndrome (JS), which is defined by the clinical triad of hypotonia, developmental delays, and a pathognomonic cerebellar and brain stem malformation referred to as a “molar tooth sign” (MTS) (Parisi & Glass, 2003).

Since its initial description in 1941 (Mohr, 1941), multiple OFDS types have been described with significant phenotypic overlap but with distinct patterns of organ system involvement and malformations (Bruel et al., 2017; Franco & Thauvin‐Robinet, 2016). OFD is inherited in an X‐linked dominant, X‐linked recessive, and autosomal recessive manner. Causal genes have not been identified for approximately 40% of OFD types (Bruel et al., 2017; Franco & Thauvin‐Robinet, 2016).

Here we describe three probands with a clinical diagnosis of OFDS VI. One patient was compound heterozygous for nonsense and frameshift *CEP164* variants, and two unrelated probands of Dominican Republic descent were homozygous for a c.29 C>A; p.(Pro10Gln)) missense variant in *TOPORS*. We propose that *CEP164* and *TOPORS* are novel causal genes for OFD VI, and suggest that the c.29 C>A allele is may represent a founder allele in the Dominican Republic population.

## 2 METHODS

### 2.1 Editorial policies and ethical considerations

All individuals and families agreed to participate in this study and signed appropriate consent forms. Permission for clinical photographs was given separately. This study was approved by the institutional IRB (Protocol # 16–013278).

### 2.2 Genetic testing methodology

Chromosomal microarray for Patient 1 was performed at SUNY Upstate Medical University. Trio exome sequencing was performed on DNA from Patient 1 at GeneDx. Prenatal chromosomal microarray and Joubert Syndrome panel testing was performed on Patient 2 at GeneDx. Trio exome sequencing was performed for Patient 2 at PreventionGenetics. Prenatal chromosomal microarray and trio exome sequencing was performed for Patient 3 at GeneDx and for his affected sibling at Integrated Genetics. Patient 3 was recruited into the Center for Applied Genomics (CAG) at CHOP for exome reanalysis. Patients 2 and 3 were matched through GeneMatcher (Sobreira et al., 2015).

### 2.3 Exome sequencing reanalysis

Under an Institutional Review Board approved protocol (Protocol # 16‐013278), informed consent was obtained from the family of Patient 3 for enrollment in CAG. Variant annotation, filtration and prioritization was performed with GDCross, a variant annotation and prioritization platform developed within CAG. Variants with ≥5x coverage were initially filtered at 0.5% gnomAD MAF and annotated with a combination of multiple tools and databases, including Variant Effect Predictor, HGMD, ClinVar, dbSNP, OMIM, HPO, PolyPhen‐2 and SIFT, and a custom‐built splice‐site annotator. The list of patient variants is filtered against the pedigree and HPO terms describing the patient's phenotype, and GDCross assigns each variant a priority score of likelihood as the causal variant for the patient's disease. Variants are ranked using a weighted combination of multiple factors, including (a) overlap with HPO terms, (b) patient and family genotypes, (c) predicted functional impact, (d) inheritance modeling, and (e) presence in mutation databases such as HGMD and ClinVar.

## 3 RESULTS

### 3.1 Patient cohort

#### 3.1.1 Patient 1

Patient 1 was the product of a naturally‐conceived pregnancy to a then 40‐year‐old G5P3➔4 mother. Family history was notable for a 12‐year‐old brother with Trisomy 21 (conceived at 27 years of age) and a 14‐year‐old sister with suspected autoimmune aplastic anemia status post bone marrow transplant. There were no medications or exposures during the pregnancy. Noninvasive prenatal testing was done due to maternal age and was normal. Twenty‐week ultrasound was also normal. An additional ultrasound was done at 29 weeks due to loss of the cervical mucus plug, and was notable for multiple congenital anomalies, prompting fetal MRI and echocardiogram, which were notable for polydactyly, brain cyst, absent vagina, and hydronephrosis. Patient was ultimately born via caesarian section at 33 weeks gestational age due to fetal tachycardia and worsening hydronephrosis. Birth weight was 3.115 kg (>97%; 50% for 36 weeks gestational age) and birth length was 43 cm (25–50%). She was admitted to the NICU for management. She was noted to have hypotonia, four‐extremity polydactyly, syndactyly of multiple digits, multiple tongue hamartomas, and urogenital sinus. A suprapubic catheter was placed with improvement in her hydronephrosis and hydrometrocolpos. Echocardiogram and ophthalmology examination were normal. Head ultrasound showed a prominent temporal horn of the left lateral ventricle. Brain MRI showed hypothalamic hamartoma and asymmetric ventriculomegaly.

**FIGURE 1:**
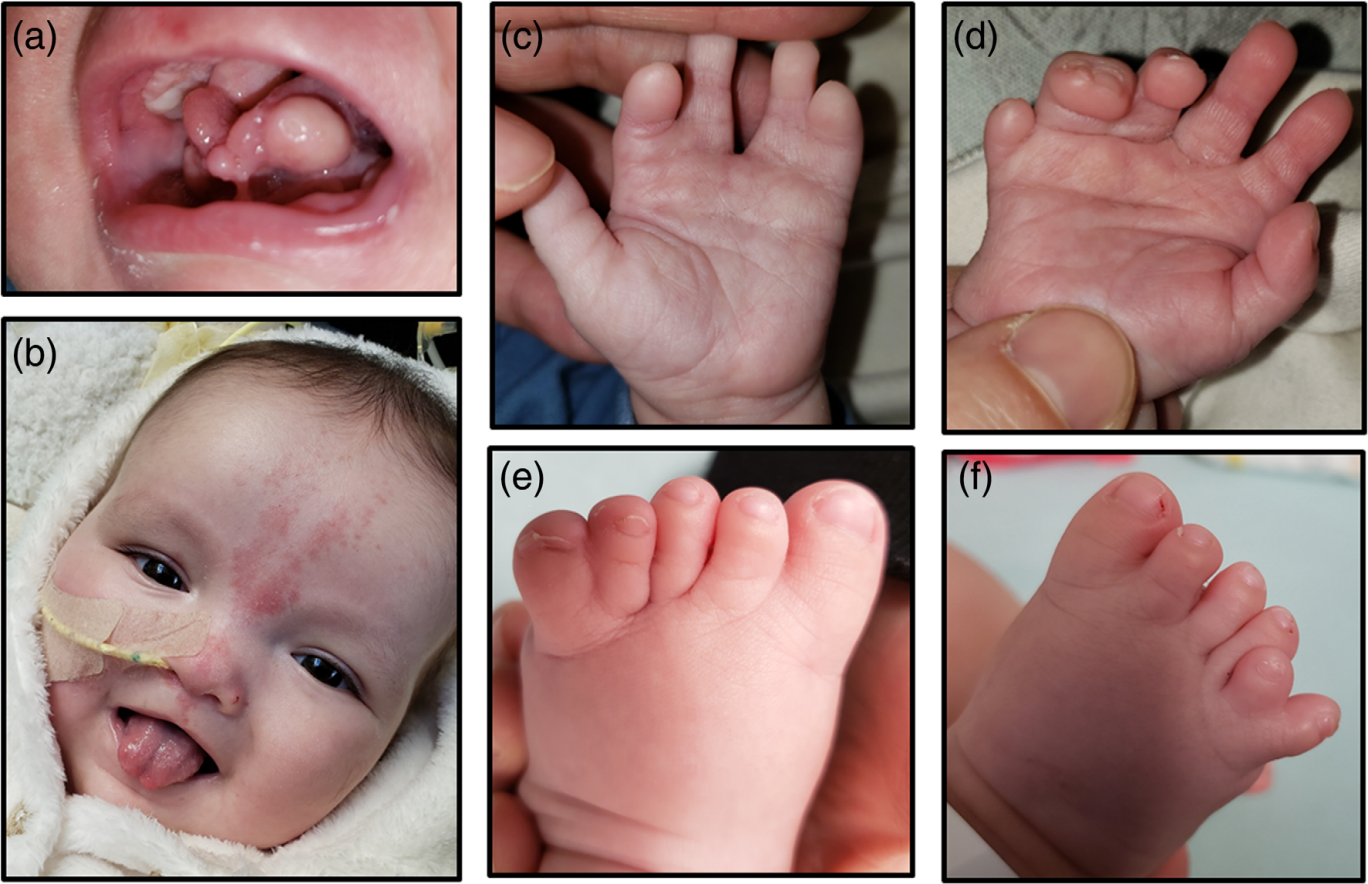
(a) Patient 1 oral cavity at 3 months of age demonstrating multiple lingual hamartomas and accessory frenula. (b) Patient 1 oral cavity at 6 months of age after hamartoma resection showing bifid tongue. Facial features notable for hypertelorism, broad nasal bridge and nevus simplex. (c) Patient 1 left hand showing 2,3 and 4,5 syndactyly. (d) Patient 1 right hand showing postaxial polydactyly and 5,6 syndactyly. (e) Left foot showing post axial polydactyly with complete 5,6 syndactyly. (f) Patient 1 right foot notable for postaxial polydactyly [Color figure can be viewed at wileyonlinelibrary.com]

She was evaluated by Genetics at 3 months of age. Growth parameters were notable for a weight of 3.65 kg (10–25% corrected), height of 48.3 cm (<3%; 50% for newborn), and head circumference of 36.5 cm (75% corrected). Physical examination was notable for hypotonia, relative macrocephaly, down‐slanting palpebral fissures, hypertelorism, broad and flat nasal bridge, upper alveolar ridge notch, multiple lingual hamartomas, accessory frenula, high palate, mild micrognathia, and linear nevus simplex extending from the forehead to the upper lip (Figure 1a,b). Extremity examination was notable for a left hand with 2,3 and 4,5 syndactyly (Figure 1c), postaxial polydactyly of the right hand with 7 digits total and complete syndactyly of digits 5,6 (Figure 1d), postaxial polydactyly with complete 5,6 syndactyly of the left foot (Figure 1e), and postaxial polydactyly of the right foot (Figure 1f).

Presence of hypothalamic hamartoma, lingual hamartomas, polydactyly, and syndactyly was concerning for OFD VI (Poretti et al., 2012). Kidney ultrasound was performed, which showed moderate, symmetric hydronephrosis with no kidney cysts. An EEG was performed, which showed intermittent focal slowing in the bifrontal region and rare epileptiform discharges in the left temporal and right occipital regions. She underwent surgical removal of her lingual hamartomas and accessory frenula, and was admitted postoperatively due to concern for silent aspiration, later confirmed on video swallow study. She is currently NPO. Patient is now 6 months of age, gestationally corrected to 4 months of age. Motor milestones are delayed with minimal head control. Social skills are normal; patient fixes and follows, smiles, and is extremely interactive.

#### 3.1.2 Patient 2

Patient 2 was born to a 31‐year‐old G4P3➔4 mother. Family history was noncontributory. Both parents were from the Dominican Republic. Pregnancy was complicated by gestational diabetes treated with metformin. Prenatal ultrasounds and follow up fetal MRI were notable for four extremity polydactyly, severely hypoplastic/absent cerebellar vermis, mildly enlarged posterior fossa, and micropenis. Patient was born via repeat caesarian section at 38 weeks gestational age. APGARS were 9 and 9 at 1 and 5 min of life, respectively. Birth weight was 3.73 kg (90%), birth length was 50.5 cm (75–90%), and head circumference was 37 cm (>97%). Patient was diagnosed postnatally with hypotonia, multiple lingual hamartomas, four‐extremity postaxial polydactyly, and micropenis. Echocardiogram demonstrated pulmonic valve stenosis, tricuspid valve regurgitation, and patent foramen ovale. MRI demonstrated arachnoid cyst and MTS.

Genetics evaluated the baby at 1 day of life and again at 4 months of life. At 4 months of life, weight was 8.9 kg (>97%; 50% for 9 months), length was 60.5 cm (25%), and head circumference was 44 cm (98%). Physical examination was notable for bilateral ptosis worse on the left, down‐slating palpebral fissures, broad nasal bridge, slightly low and posteriorly‐rotated ears, multiple lingual hamartomas, 2/6 murmur, and hypotonia with significant head lag. Patient did not fix or follow. Kidney ultrasound was normal, and ophthalmology examination was performed, but the results are unknown. Patient is currently 10 months of age. Clinical course is complicated by severe developmental delay.

#### 3.1.3 Patient 3

Patient 3 was the product of a naturally‐conceived pregnancy to a then 21‐year‐old G2P0➔1 mother. Both parents were from the Dominican Republic. Family history was notable for consanguinity with the mother's maternal grandfather and the father's paternal grandmother being siblings (second‐cousin union) and for a prior pregnancy terminated for multiple congenital anomalies, including lemon‐shaped head with frontal bossing, posterior fossa cyst, encephalocele, and arthrogryposis with bilateral talipes equinovarus. Microarray and exome sequencing were nondiagnostic.

Pregnancy was complicated by prenatal ultrasound concerning for multiple congenital anomalies, prompting fetal MRI, which demonstrated bilateral ventriculomegaly, dandy‐walker malformation, occipital meningocele, ambiguous genitalia, bilateral talipes equinovarus, and shortened long bones. Patient was born at 36 weeks gestational age via caesarian section for fetal macrocephaly. APGARs were 2 and 8 and 1 and 5‐min of life, respectively. Birth weight was 3.17 kg (75%), birth length was 45 cm (20%), and head circumference was 42 cm (>97%). He was diagnosed postnatally with cleft palate and four‐extremity polydactyly. He developed respiratory distress in the delivery room requiring positive pressure ventilation, and was intubated shortly thereafter for persistent apneas and desaturations.

He was evaluated by Genetics on day of life 3. Physical examination was notable for macrocephaly with prominent forehead and occiput, down‐slanting palpebral fissures, hypertelorism, nasal milia, bifid tongue, lingual hamartomas, micrognathia, four‐extremity postaxial polydactyly, micropenis and hypotonia. Due to concern for OFDS, an abdominal ultrasound, echocardiogram, brain MRI and ophthalmology examination were requested. Abdominal ultrasound showed structurally normal liver and kidneys, echocardiogram showed a structurally normal heart, ophthalmology examination showed bilateral optic nerve colobomas, and brain MRI showed dandy‐walker malformation, left parasagittal occipital encephalocele, possible ectopic posterior pituitary gland, possible left frontal lobe cleft, and small optic nerves.

Patient is currently 8‐months of age. Growth parameters are notable for a weight of 10.5 kg (95%), a height of 61 cm (<3%; 50% for 2.5 months of age), and a head circumference of 46 cm (>97%). Physical examination is largely unchanged. Patient's clinical course has been complicated by hydrocephalus status post shunt placement complicated by multiple shunt infections, respiratory failure status post tracheostomy placement, G‐tube dependence, cortical visual impairment, neuro‐irritability, spasticity, central adrenal insufficiency, and profound developmental delay.

### 3.2 Genetics testing

#### 3.2.1 Patient 1

Patient 1 had a chromosomal microarray performed, which showed a 470 base pair duplication of unclear clinical significance at 5q35.2 (hg19: 175,668,563–176,138,247) including the *NOP16*, *FAF2*, *SIMC1*, *CLTB*, and *SNCB* genes. Parental testing was declined. Trio exome sequencing was performed at GeneDx, and was notable for a paternally‐inherited pathogenic *CEP164* frameshift variant (c.2535_2536dupGG; p.Glu846Glyfs*10) and a maternally‐inherited pathogenic *CEP164* nonsense variant (c.3055 C>T; p.Gln1019*).

#### 3.2.2 Patient 2

Patient 2 had a prenatal microarray and JS gene panel performed at GeneDx, which were nondiagnostic. Trio exome sequencing was performed postnatally at PreventionGenetics, and was notable for biallelic missense variants of uncertain significance in the candidate gene *TOPORS* (c.29 C>A; p.Pro10Gln) and a 6 Mb region of homozygosity on chromosome 9 (hg19: 32,425,909–38,423,826) including the *TOPORS* gene.

#### 3.2.3 Patient 3

Patient 3 had a prenatal microarray and trio exome sequencing performed at GeneDx. Microarray was notable for two regions of homozygosity on chromosomes 8 (hg19: 128,664,940–140,805,582) and 9 (hg19: 22,920,663–40,087,758) but no copy number variations. Exome sequencing was notable for a paternally‐inherited pathogenic variant in *PMM2* (c.713G>A; p.(Arg238His)), associated with the autosomal recessive congenital disorder of glycosylation PMM2‐CDG. Microarray and trio exome sequencing were also performed on the previously affected fetus. Microarray was notable for two regions of homozygosity on chromosomes 7 (hg19: 44,098,864–58,019,983) and 9 (hg19: 25,000,261–40,087,758). Patient and family were enrolled in CAG for research exome reanalysis given negative clinical testing. Reanalysis revealed biallelic variants of uncertain significance in *TOPORS* (c.29 C>A; p.Pro10Gln) in the affected proband. These variants were clinically confirmed via gene sequencing at PreventionGenetics. Integrated Genetics subsequently confirmed that the affected fetus also harbored the *TOPORS* variants. The Pro10Gln variant was subsequently upgraded to likely pathogenic for both patients by PreventionGenetics given the similar clinical presentations and identical variants. Of note, *TOPORS* maps to the region of homozygosity shared by both children.

## 4 DISCUSSION

Oral facial digital syndromes (OFDS) are a group of rare ciliopathy syndromes characterized by the core features of oral cavity malformations, facial dysmorphisms, and digit anomalies (Bruel et al., 2017; Franco & Thauvin‐Robinet, 2016). OFDS subtypes are classified based on pattern of associated features such as structural brain differences, intellectual disability, skeletal differences, congenital heart disease and kidney disease, with over 18 subtypes described to date (Table 1).

**TABLE 1:** Tabulated view of phenotypes associated with 14 different OFD syndromes as compared to the phenotypes of our described patients [Color table can be viewed at wileyonlinelibrary.com]

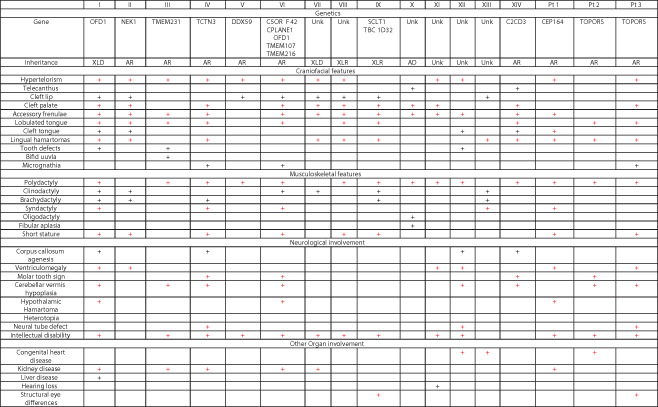

*Notes*: Overlapping features are highlighted in red. Given the hypertelorism, lingual hamartomas, tongue lobulations, polysyndactyly, hypothalamic hamartoma, neural tube defects, molar tooth sign and intellectual disability seen in our patients, we propose that our patients' phenotypes are most consistent with OFD VI.

**FIGURE 2:**
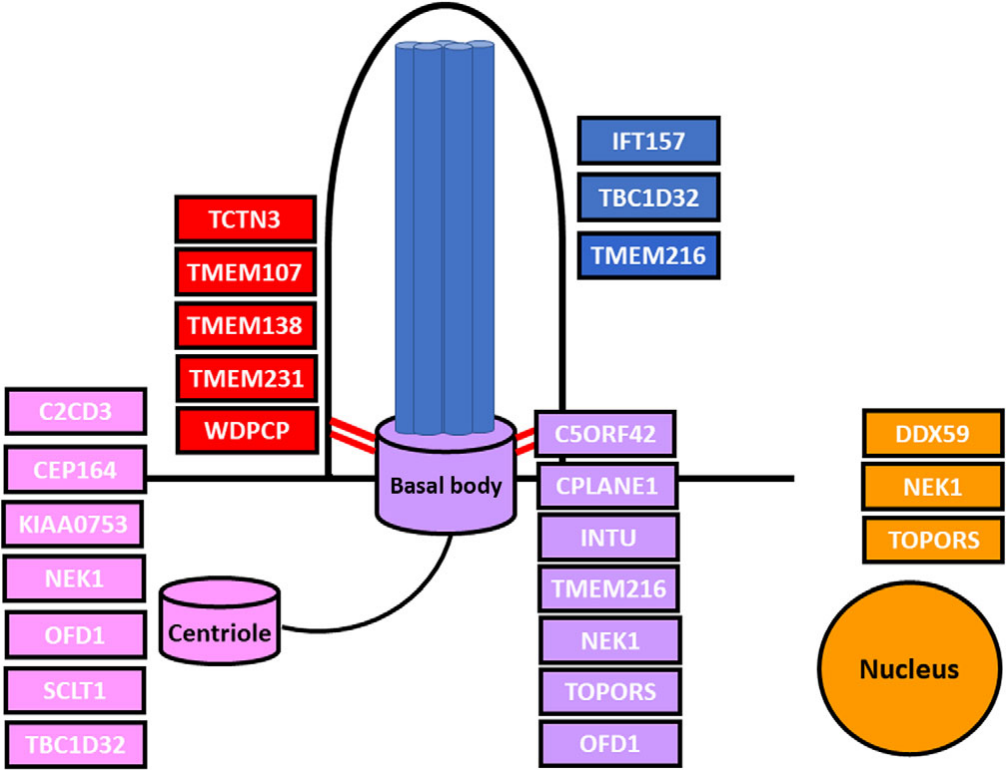
Genes associated with OFDS localize to multiple ciliary sub‐compartments. The primary cilium is anchored to the cell by the basal body (purple), which is formed from the mother centriole and remains associated with its centriole pair (pink). The transition zone (red) serves as the gatekeeper for the cilium to control entry and exit. The axoneme (blue) is composed of microtubule doublets that provides structure to the cilium and also forms the framework for ciliary transport. The nucleus (orange) is an important target of ciliary signaling (EHZ) [Color figure can be viewed at wileyonlinelibrary.com]

Over 16 genes have now been identified as causal for OFDS (Bruel et al., 2017). These genes encode components of the primary cilium, a mechanosensory organelle that facilitates organ growth and patterning (Berbari et al., 2009; Fry et al., 2014). OFDS genes localize to virtually every ciliary subsegment, including the basal body, centriole, transition zone, and axoneme (Figure 2). The exact mechanism by which ciliary dysfunction causes the clinical features of OFDS is unknown.

Though initially described 80 years ago, up to 40% of the described OFDS subtypes do not have an associated gene and many patients with a clinical diagnosis of OFDS have negative molecular testing, highlighting our incomplete understanding of the genetic landscape of these conditions (Bruel et al., 2017; Franco & Thauvin‐Robinet, 2016). Additionally, many genes associated with OFDS are also associated with other ciliopathy syndromes, most commonly JS; however, genotype–phenotype correlation remains elusive, and it is difficult to use mutation type to predict which ciliopathy phenotype a patient will ultimately have (Lambacher et al., 2016; Shaheen et al., 2013; Valente et al., 2010).

Here we describe two novel genes identified in patients with clinical features consistent with OFD VI: *CEP164*, previously identified as causal for the ciliopathies nephronophthisis, JS and Bardet Biedl syndrome, and *TOPORS*, a gene associated with the autosomal dominant ciliopathy RP, but never before with syndromic ciliopathy.

*CEP164* maps to 11q23.3 and encodes centrosomal protein of 164 kDa, a component of the centriolar distal appendage. CEP164 is required for vesicular docking at the centriole, thereby enabling ciliogenesis (Graser et al., 2007). CEP164 has also been associated with coordination of the DNA damage response (Pan & Lee, 2009; Sivasubramaniam et al., 2008); however, this has recently been challenged (Daly et al., 2016). Morpholino‐mediated *cep164* knockdown in zebrafish disrupts the DNA damage pathway and causes nephronophthisis‐spectrum disease and hydrocephalus (Chaki et al., 2012). Total body *cep164* deficiency is embryonic lethal in mice, associated with holoprosencephaly, abnormal heart development, and gross patterning defects, and conditional *cep164* deficiency in multi‐ciliated cells causes hydrocephalus, lung disease, and infertility, supporting a role for *CEP164* in ciliary function (Siller et al., 2017). Biallelic *CEP164* variants have been identified in multiple individuals affected by ciliopathy‐spectrum disease, including Leber congenital amaurosis, Bardet‐Biedl syndrome, and JS with associated features including obesity, bronchiectasis, liver fibrosis, and intellectual disability (Chaki et al., 2012; Shamseldin et al., 2020). Pathogenic *CEP164* variants are believed to cause ciliopathy‐spectrum disease by disrupting ciliogenesis and possibly also through modulation of the DNA damage response and cell cycle control (Chaki et al., 2012; Graser et al., 2007; Pan & Lee, 2009; Sivasubramaniam et al., 2008). We hypothesize that Patient 1 presented with severe ciliopathy‐spectrum disease because her nonsense and frameshift *CEP164* variants more significantly impair *CEP164* function. Indeed, patients with biallelic nonsense *CEP164* variants presented with Bardet‐Biedl and JS phenotypes, as opposed to patients harboring missense variants, who presented with nephronophthisis and retinal disease (Chaki et al., 2012; Shamseldin et al., 2020).

*TOPORS* maps to 9p21.1 and encodes topoisomerase I‐binding arginine/serine rich protein, a ubiquitously‐expressed protein that localizes to the nucleus and basal body (Chakarova et al., 2011). *TOPORS* was originally identified in a screen for topoisomerase interacting proteins, and was later found to also interact with p53 (Haluska Jr et al., 1999; Zhou et al., 1999). *TOPORS* functions as an E3 ubiquitin ligase, and plays a role in protein ubiquitination and sumoylation (Pungaliya et al., 2007; Rajendra et al., 2004). Morpholino‐mediated *topors* knockdown in zebrafish causes microphthalmia, kinked tail and body edema, all established ciliopathy phenotypes in fish (Chakarova et al., 2011). Heterozygous *TOPORS* variants are associated with the retinal ciliopathy retinitis pigmentosa (RP) (Bowne et al., 2008; Chakarova et al., 2007).

**FIGURE 3:**
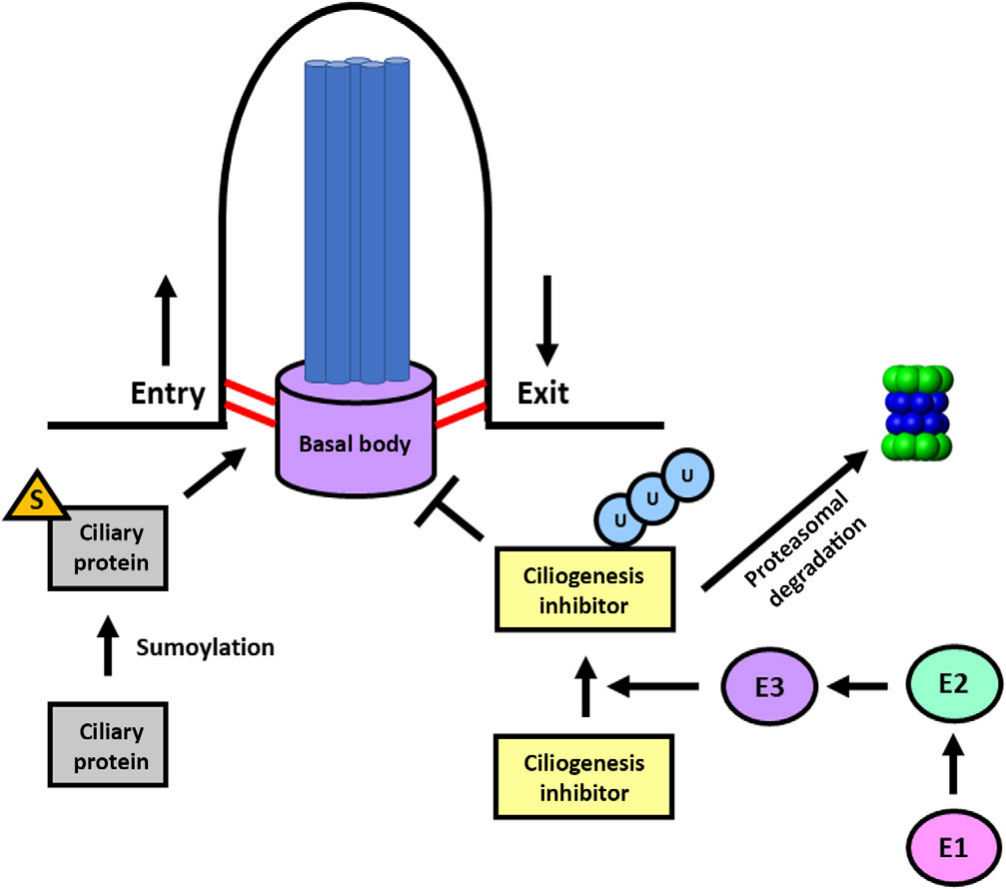
Proposed mechanism for TOPORS‐spectrum disease. Center: The cilium is a hair‐like appendage that exists atop most cell types. It consists of a basal body (purple), a modified centriole, which anchors the microtubules that make up the axoneme (blue) to the cell. The transition zone (red) at the ciliary base controls entry into the cilium. Right: E1 (pink), E2 (green), and E3 (purple) facilitate the transfer of ubiquitin to appropriate substrates to control ciliogenesis and ciliary function. Left: Many ciliary proteins undergo post‐translation sumoylation to control their ciliary entry. TOPORS is an E3 ubiquitin ligase that also has sumoylation activity. We hypothesize that TOPORS deficiency disrupts ciliary function by interfering with ciliary protein ubiquitination and possibly sumoylation [Color figure can be viewed at wileyonlinelibrary.com]

The mechanism by which heterozygous *TOPORS* variants cause disease is unknown. Biallelic *TOPORS* variants have not been previously reported. Given the localization of TOPORS at the ciliary base and in the nucleus, loss of *TOPORS* function could cause ciliopathy‐spectrum disease by disrupting ciliary homeostasis directly or through dysregulated gene expression. We hypothesize that loss of *TOPORS* function disrupts ciliogenesis and ciliary function by interfering with the proper ubiquitination and turnover of ciliary proteins and morphogens (Figure 3). Disruption of the ciliary ubiquitin‐proteasome system disrupts ciliogenesis, and biallelic variants in genes involved in regulation of the ubiquitin‐proteasome system have been identified in ciliopathy patients (Gerhardt et al., 2015; Izawa et al., 2015; Kasahara et al., 2014).

All three *TOPORS* patients harbor identical biallelic missense variants (c.29 C>A; p.Pro10Gln) in *TOPORS*. *TOPORS* is intolerant to loss‐of‐function variants, with a pLI of 1. The Pro10Gln variant has been reported in gnomAD and ExAc at low allele frequency, and there are no homozygotes. In silico prediction tools such as polyphen and SIFT support pathogenicity. Given the overlapping patient phenotypes, the Pro10Gln variant was reclassified by PreventionGenetics as likely pathogenic. Interestingly, neither parent nor any relatives report vision difficulties or RP, suggesting that this variant is a hypomorph, insufficient to cause retinal disease in the heterozygous state, but severe enough to cause syndromic ciliopathy in the homozygous state.

Combined autosomal dominant and autosomal recessive inheritance has been reported for ciliopathies. Heterozygous *PKD1* variants are the most common cause of adult‐onset autosomal dominant polycystic kidney disease, while biallelic *PKD1* variants are associated with early and potentially neonatal‐onset, severe polycystic kidney disease (Al‐Hamed et al., 2019; Audrézet et al., 2016; Durkie et al., 2021). Heterozygous *DNAJB11* variants are associated with autosomal dominant polycystic kidney disease, while biallelic variants cause Ivemark II syndrome or renal‐hepatic‐pancreatic dysplasia syndrome (Jordan et al., 2021). We propose that *TOPORS* has similar properties, with heterozygous variants causing the milder, organ‐specific ciliopathy retinitis pigmentosa, and biallelic variants causing OFDS‐spectrum syndromic ciliopathy.

Our identified *TOPORS* patients both presented with the clinical stigmata of OFDS, including oral cavity malformations, dysmorphic features, and polydactyly; however, Patient 2 also presented with MTS, which is pathognomonic for JS. The overlap between OFDS and JS is well established, with several reports of individuals harboring features of both syndromes (Aljeaid et al., 2019; Bhardwaj et al., 2018; Johnston et al., 2017; Wentzensen et al., 2015). Several genes are associated with both conditions, including *C5ORF42*, *OFD1*, *TCTN3*, *TMEM107*, *TMEM216*, and *TMEM231*, and JS and OFDS can be allelic to each other (Bruel et al., 2017; Coene et al., 2009; Franco & Thauvin‐Robinet, 2016). We propose that *TOPORS* also bridges OFDS and JS. It is unknown at this time whether this *TOPORS* phenotype is specific to the Pro10Gln variant identified in our probands, or whether it can be generalized to other gene variants. It is also unclear why Patient 3 and his affected sibling presented with neural tube defects while Patient 2 presented with MTS. Of note, both families trace their ancestry to the Dominican Republic, suggesting a possible founder allele.

In conclusion, we report on two novel genes associated with OFD VI, *TOPORS* and *CEP164*, further expanding the genetic differential of OFDS. Our cases highlight the phenotypic and genetic diversity of this family of diseases, and again implicate the ubiquitin‐proteasome system in ciliary disease.

## CONFLICT OF INTEREST

None.

## AUTHOR CONTRIBUTIONS

Alanna Strong conceptualized and designed the study, evaluated Patients 1 and 3 clinically, helped in variant interpretation and data analysis, and drafted the manuscript. Laurie Simone and Helio Fernando Pedro helped evaluate Patient 2 and helped analyze phenotypic and genetic data from all patients. Hayley Ron and Jennifer Kalish helped evaluate Patient 3 and guided the phenotypic workup. Elaine Zackai helped evaluate Patient 3, guide the phenotypic workup and interpret the genetic variants identified. Anthony Krentz helped analyze the exome for Patient 2. Deborah Watson and Courteney Vaccaro facilitated genetic data collection and analysis for Patient 3. Hakon Hakonarson helped conceptualize and design the study, contributed to data analysis, critically reviewed and edited the manuscript, and provided funding for the work. All authors approved the final manuscript as submitted and agree to be accountable for all aspects of the work.

## Data Availability

Data sharing is not applicable to this article as no new data were created or analyzed in this study.
